# Laparoscopic uteroovarian ligament truncation and uterosacral oophoropexy for idiopathic recurrent ovarian torsion: case report and review of literature

**DOI:** 10.1186/2054-7099-1-2

**Published:** 2015-04-21

**Authors:** Paula C Brady, Aaron K Styer

**Affiliations:** 3 Vincent Department of Obstetrics and Gynecology, Massachusetts General Hospital, Harvard Medical School, 55 Fruit St, Boston, MA 02114 USA; 4 Vincent Reproductive Medicine and IVF, Massachusetts General Hospital, Harvard Medical School, Yaw 10A, 55 Fruit Street, Boston, MA 02114 USA

**Keywords:** Torsion, Recurrent, Oophoropexy, Uteroovarian ligament fixation, Laparoscopy

## Abstract

**Background:**

Unilateral recurrent ovarian torsion in adults is unusual following treatment of common underlying risk factors (e.g. benign cysts). Subtle anatomic etiologies, such as an elongated uteroovarian ligament and robust ovarian volume, are commonly underappreciated and may contribute to idiopathic recurrent unilateral torsion in adults. As seen in this case, combined surgical procedures may be required to prevent recurrence.

**Case:**

28 year old nulligravid woman with seven episodes of right ovarian torsion (without adnexal pathology)—six of those within 18 months—refractory to a series of previous surgical interventions. Laparoscopic uteroovarian ligament truncation with interval uterosacral ligament oophoropexy was employed. Ovarian torsion has not occurred in 45 months.

**Conclusion:**

Uteroovarian ligament truncation and uterosacral ligament oophoropexy is a feasible and effective combined surgical approach for the prevention of recurrent idiopathic ovarian torsion in adults without obvious risk factors.

## Background

Ovarian torsion comprises approximately 3% of all gynecologic surgical emergencies, and occurs predominately in women of childbearing age [1]. The condition occurs following twisting of the ovary on the main vascular pedicle (infundibulopelvic ligament [IPL] containing the ovarian artery and vein), which results in impedance of venous outflow with continued arterial inflow, and eventually leads to congestion, ischemia, and progressive necrosis with prolonged torsion. In adults, it is usually associated with benign ovarian masses (particularly masses greater than 5 cm) [2]. Other common risk factors include ovulation induction and pregnancy. Right torsion is twice as common as left torsion, and is thought to be due to limitation of left ovarian mobility by the sigmoid colon [3].

In contrast to adults, approximately 50% of cases of torsion in pediatric and adolescent patients are idiopathic and do not have discernible risk factors [3]. Many clinicians suspect that a congenitally elongated and lax uteroovarian ligament (UOL) and IPL result in ovarian hypermobility in these circumstances [3]. Additionally, patients with polycystic ovarian syndrome (PCOS), who may have enlarged ovaries associated with hyperandrogenism and multicystic ovaries, may be at increased risk for torsion [4]. In a recent review of six pediatric patients with unilateral torsion, Shah *et al*. observed enlarged contralateral ovaries meeting criteria for PCOS in four of five patients with no other identifiable cause for torsion [4]. While no studies have formally identified PCOS as a risk factor for torsion, case reports and small case series in pediatric patients suggest screening adolescents with ovarian torsion for evidence of PCOS [4].

Unfortunately, the vast majority of recommendations for the prevention of recurrent idiopathic torsion are derived from case series in the pediatric adolescent population. Due to the rarity of this condition in adults, comparative trials of surgical approaches are not available. This case report illustrates an extraordinary case of recurrent idiopathic ovarian torsion in an adult refractory to several prior procedures, and describes the novel approach of combined procedures to prevent recurrence.

## Case presentation

28-year-old nulligravid woman with a history of PCOS was referred to discuss options for the prevention of recurrent unilateral ovarian torsion. From May 2002 through February 2010, she had five episodes of symptomatic right ovarian torsion necessitating laparoscopic ovarian detorsion and oophoropexy at several institutions at progressively shorter intervals of time, with four months between each of the last three episodes.

Her surgical history includes:

2002: Laparoscopic right ovarian detorsion. Ovarian torsion two times around the IPL was noted.

2008: Laparoscopic right ovarian detorsion. Ovarian torsion four times around the IPL was observed. Right oophoropexy was performed with suture ligation of the right ovary to the right round ligament with a single interrupted absorbable suture (0-Vicryl, Ethicon, Somerville, NJ, USA) and standard intracorporeal knots (IK).

2009: Laparoscopic right ovarian detorsion. Ovarian torsion two times around the IPL was observed. Right oophoropexy with fixation to the right uterosacral ligament (USL) was performed with a single interrupted absorbable suture (2–0 PDS, Ethicon, Somerville, NJ, USA) and standard extracorporeal knots. No residual right round ligament was visualized.

2009: Laparoscopic right ovarian detorsion. Ovarian torsion five times around the IPL was observed.

2010: Laparoscopic right ovarian detorsion. Ovarian torsion four times around the IPL was observed. An “elongated” right UOL was observed.

During consultation, physical examination was unremarkable. Pelvic ultrasound revealed bilateral ovarian volume > 10 cm^3^ with central stromal prominence and peripheral follicular distribution consistent with previously described morphology associated with PCOS [5]. Because of the multiple prior failed ovarian fixation/oophoropexy procedures and the most recent intraoperative finding of an “elongated” UOL, an additional laparoscopic procedure with right UOL truncation was planned.

**Figure 1:**
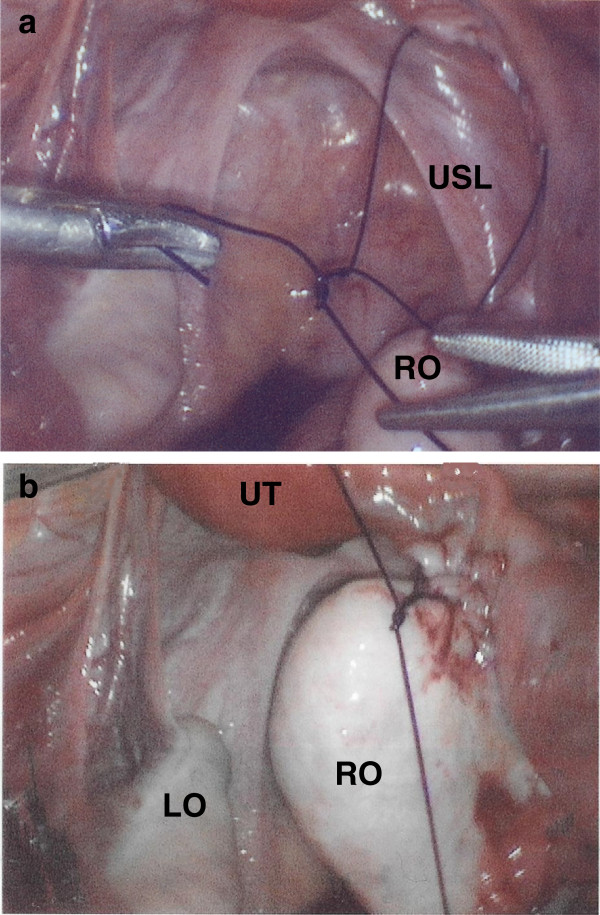
**Laparoscopic right uteroovarian ligament (UOL) truncation.** RO: right ovary, LO: left ovary, UT: uterus, USL: uterosacral ligament.

During intraoperative visualization, *in situ* asymptomatic right ovarian and fallopian tube torsion six times around the IPL was observed. No gross evidence of ischemia or adnexal pathology was observed. The right UOL ligament was 6 cm in length with an atrophic caliber. An uncomplicated elective laparoscopic right ovarian detorsion and right UOL truncation was performed. A single continuous running absorbable suture (0 PDS, Ethicon Inc. Somerville, NJ, USA) was initiated at the right UOL attachment to the right ovary with sequential bites spaced 1 cm apart and ending at a point 5 mm proximal to the site of the uterine insertion of the UOL (Figure 1a). The suture was then tied with standard IK in order to restore normal length to 2 to 3 cm (Figure 1b). Chromopertubation was performed and bilateral fill and free spill was observed in the right fallopian tube.

One month following UOL truncation, she presented with 12 hours of severe right lower quadrant pain and persistent nausea. During evaluation, she relayed that her symptoms were similar to prior episodes of ovarian torsion. Laboratory evaluation and pelvic ultrasound were unremarkable. Physical examination revealed right lower quadrant abdominal pain, rebound tenderness without guarding, and exquisitely tender right adnexa. Given her presentation and prior history of recurrent right torsion with failed ovarian fixation procedures, she underwent diagnostic laparoscopy. Intraoperative findings included: right ovarian torsion one time around the IPL, with a normal length right UOL. The pelvis was otherwise unremarkable. An uncomplicated laparoscopic right ovarian detorsion was performed.

**Figure 2:**
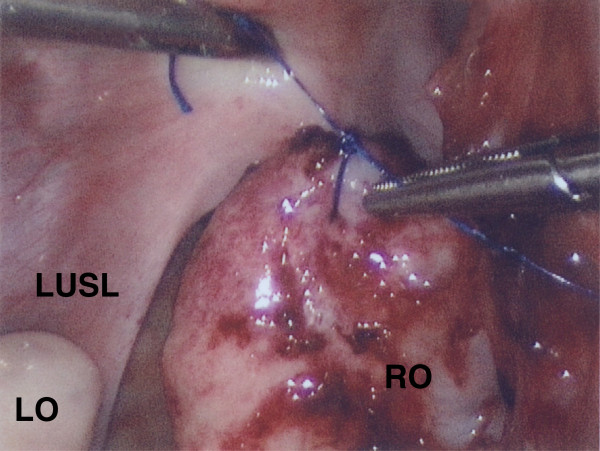
**Laparoscopic right uterosacral ligament (USL) fixation/oophoropexy.** RO: right ovary, UT: uterus, LUSL: left uterosacralligament.

In light of the unanticipated recurrent ovarian torsion for the seventh time and the recent surgical truncation of the right UOL to physiologic length, she subsequently underwent a laparoscopic right oophoropexy with fixation to the right USL. Fixation was accomplished with two individual interrupted permanent sutures (2–0 Proline, Ethicon, Somerville, NJ, USA) with attachment of the antimesenteric aspect of the ovarian apex (sutures at cortical depth of 5 mm at the ovarian pole opposite to insertion of uteroovarian ligament) to the apex of the right USL, and attachment of the lateral aspect of the ovary 1 cm inferior to ovarian apex to the mid right USL in order to ensure an axial orientation of the ovary in the pelvis and placement in the right ovarian fossa (Figure 2). Permanent suture was utilized given failure of the prior USL oophoropexy with absorbable suture. Since this procedure, she has done well and has not had any additional episodes of right ovarian torsion in the past 45 months. An interval pelvic ultrasound was performed 4 weeks postoperatively and revealed appropriate Doppler flow, with unchanged ovarian volume and follicular distribution.

## Conclusions

Although recurrence of unilateral ovarian torsion is less common in adults than in children, the rate of recurrence may be increased in adults in whom no cyst(s) or other identifiable risk factor(s) are identified [6]. Preventive measures have been recommended for recurrent torsion in adolescents, particularly in PCOS patients with polycystic ovarian morphology, with robust ovarian volume (>10 cm^3^) [6]. However, due to the rarity of recurrence in adults, oophoropexy is not universally recommended or performed during the first or second episode of torsion [7]. Overall, there is a lack of consensus on indications and approaches for surgical prevention of recurrent torsion in adults.

Techniques for the prevention of recurrent torsion include UOL truncation or oophoropexy. UOL truncation involves surgical reduction of the UOL to normal physiologic length. Oophoropexy may involve fixation of the ovary to the pelvic sidewall, to the ipsilateral round ligament, to the posterior aspect of the uterine fundus, or to the USL, with either absorbable or permanent sutures [8]. UOL truncation may be theoretically preferable to USL oophoropexy, as this approach may restore normal anatomic length (in the case of an elongated UOL), while oophoropexy poses hypothetical concerns for excessive tension on the adnexa with impairment of fallopian tube function [7, 8]. Although both approaches are considered reasonable options, there are no randomized studies comparing recurrence rates or long-term fertility effects of either technique. Favorable pregnancy outcomes have been demonstrated with both approaches with absorbable and nonabsorbable suture [7]. Unfortunately, there is no consensus in the literature regarding use of absorbable or nonabsorbable suture. Two investigators recommend nonabsorbable suture in light of their experience with recurrence when using absorbable suture. Conversely, one case of ovarian atrophy among 9 patients was reported after use of nonabsorbable suture [7, 9, 10]. Of note, in this case, the definitive intervention employed nonabsorbable suture, without evidence of ovarian compromise by ultrasound one month later.

This patient’s unique clinical course spans the continuum of both adult and pediatric gynecology. Her potential risk factors for recurrent torsion—robust ovarian volume and elongated right UOL—have been most commonly identified in pediatric and adolescent populations. Furthermore, the methods of UOL truncation and USL oophoropexy utilized in this case have been predominately reported in the pediatric surgical literature. Notably, this case highlights the significance of these subtle and underappreciated anatomic derangements in adults, particularly relevant in “idiopathic” cases. Furthermore, the combination of UOL truncation with interval USL oophoropexy with nonabsorbable suture ultimately halted the accelerating course of recurrent torsion in this patient. This case demonstrates that adult patients with idiopathic recurrent torsion which is refractory to single surgical intervention, may benefit from a combined surgical approach. Simultaneous UOL truncation and USL oophoropexy is a reasonable option for adult women with recurrent idiopathic unilateral ovarian torsion (especially with an elongated UOL) without overt risk factors for torsion and who desire future fertility.

## Consent

Written informed consent was obtained from the patient for publication of this Case report and any accompanying images. A copy of the written consent is available for review by the Editor-in-Chief of this journal.

## Authors’ information

P.C.B. is a resident physician in the Brigham and Women’s Hospital/Massachusetts General Hospital Integrated Residency Program in Obstetrics and Gynecology, affiliated with the Harvard Medical School.

A.K.S. is a reproductive endocrinologist and reproductive surgeon, and an Assistant Professor in Obstetrics, Gynecology and Reproductive Biology at Harvard Medical School and serves as the Associate Director for the Basic Science Research Program in the Reproductive Endocrinology and Infertility Fellowship in the Vincent Department of Obstetrics and Gynecology at the Massachusetts General Hospital.
